# Influences of Environmental Factors on Leaf Morphology of Chinese Jujubes

**DOI:** 10.1371/journal.pone.0127825

**Published:** 2015-05-28

**Authors:** Xiaopeng Li, Yupeng Li, Zhong Zhang, Xingang Li

**Affiliations:** 1 College of Forestry, Northwest A&F University, Yangling, Shaanxi, China; 2 Research Centre for Jujube Engineering and Technology of State Forestry Administration, Northwest A&F University, Yangling, Shaanxi, China; 3 College of Water Resources and Architectural Engineeing, Northwest A&F University, Yangling, Shaanxi, China; Beijing Forestry University, CHINA

## Abstract

Rainfall and temperature are the primary limiting factors for optimum quality and yield of cultivated jujube (*Ziziphus jujuba* Mill.). Adaptation to arid and cool environments has been and remains an important goal of many jujube improvement programs. This study summarized the survey results of 116 Chinese jujube varieties grown at 33 sites in China. The objective was to identify the environmental factors that influence leaf morphology, and the implications for breeding and introduction of new jujube varieties. Jujube leaf morphological traits were evaluated for their potential relationships with mean annual temperature (MAT) and mean annual precipitation (MAP). The results showed that many leaf morphological traits had a strong linear relationship with local precipitation and temperature. Longer veins per unit area (VLA) and reduced leaf area and leaf perimeter were typical of arid areas. VLA was inversely related to MAT and MAP at the centers of origin of jujube. There was a positive relationship between leaf shape (perimeter^2^/area) and both MAT and MAP. These results indicated that leaf vein traits of Chinese jujubes might have resulted from their adaptation to environmental factors in the course of long-term evolution. Principal component analysis allocated the 116 jujube varieties to three different groups, differentiated on the basis of morphological and physiological leaf characteristics. Jujube varieties from the Hebei, Shandong, Henan, southern Shanxi and central Shaanxi provinces were closely related, as were varieties from northwest Shanxi and northeast Shaanxi provinces, and varieties from the Gansu and Ningxia provinces. These close relationships were partially attributed to the frequent exchanges of varieties within each group. Leaf venation characteristics might be used as reference indices for jujube variety introduction between different locations.

Influences of Environmental Factors on Leaf Morphology of Chinese Jujubes

## Introduction

Cultivated jujube (*Ziziphus jujuba* Mill.), which belongs to the Rhamnaceae family, is an economically important fruit tree in China [1]. Jujube fruits are consumed for their medicinal value [2].There are approximately 700 jujube varieties in existence. In China, the earliest jujube cultivation and domestication occurred about 7000 years ago along the Yellow River Canyon in the North Shaanxi and Shanxi provinces. Thus, this area is usually considered as the jujube origin center [1,3].

The process of jujube domestication has been linked to human selection and natural reproduction [3]. During the long history of evolution, Chinese jujube has become greatly differentiated [4]. Jujube is diverse for fruit shape (round, flat round, oblong, columned, ovate, inverse ovate, olive-like, and red-pepper-like), fruit taste (very sweet, sweet, acid, sweet-acid, and acid-sweet), seed (plump, shriveled, and seedless), stipular spines (strong, weak, and absent), and so on [5]. However, the wide distribution and domestication of jujube has led to some problems, which have impeded breeding and the introduction of improved jujube varieties [4,6].

Chinese jujube is a deciduous fruit tree, typically possessing thorny branches. Their leaves are ovate-acute, with three conspicuous veins at the base and finely toothed margins [7]. They can withstand extreme arid conditions and produce reasonable yields. Many famous Chinese jujube cultivars are cultivated in Northwest China, which is well known for its arid climate. In Northwest China, the annual precipitation is usually below 200 mm in arid, 200–450 mm in semi-arid, and 450–650 mm in sub-humid regions [1]. Jujube can grow and thrive in a wide range of temperatures. Usually it could tolerate cold winters and survive temperatures as low as −20°C. This enables jujube to grow in mountains or deserts, and in cold regions [8]. In addition, under different climatic conditions, jujube cultivars are diverse for traits, such as fruit shape, flavor, color, botany traits, and propagation ability [4,9,10].

Leaf traits can influence fitness of trees through biochemical, physiological, morphological, or developmental mechanisms [11]. A number of studies have demonstrated strong linear relationships between vein traits of various plant species and their hydraulic, photosynthetic, anatomical, and compositional traits, which generally influence flux of water and carbon into and out of leaves [12,13]. Leaf veins transport substances and provide mechanical stability [14]. Plant functional traits have adapted to environments through long-term evolution, embodying the ecological strategies of plants under selective pressure in natural conditions. The leaves of terrestrial plants are highly diverse and display variable leaf venation patterns [14], while the highly diverse leaf function is mirrored by highly diverse in venation network geometry [15]. Leaves are also the major organs of photosynthesis in jujube plants. Photosynthesis of jujube is highly sensitive to water deficit, which directly affects development and productivity [16]. Hence, the relationship between functional leaf traits and drought and cold tolerance must be understood to obtain high yield and good quality jujube production.

In recent years, the influences of leaf hydraulic traits on leaf and plant-level functions have gained increasing attention [12,17,18]. According to the ‘flux trait network’ hypothesis, leaf traits and plant performance are interrelated, with a key role played by structural and physiological variables that influence fluxes [17–19]. Veins are a major component of the pathway of leaf water transport [20]. Vein traits are important in determining hydraulic conductance, which in turn is related to stomatal conductance (gs) and photosynthetic rate per leaf area (A_area_). Both gs and A_area_ influence photosynthetic rate per leaf mass (A_mass_) and plant relative growth rate [18]. Vein traits, such as vein length per unit area (VLA), play a crucial role in leaf gas exchange and plant growth [21]. VLA influences hydraulic conductance, stomatal conductance, and photosynthetic rate [19,22]. The ratio of VLA to leaf hydraulic conductance (K_leaf_) represents the capacity for leaf water transport per leaf area per unit water potential [17]. VLA is typically negatively and linearly related to leaf size, as major veins appear early in leaf development, and are spaced apart during leaf expansion [23].

Higher plants typically cope with varying environmental conditions through changes in their tissues and organs [24]. Plants respond to environmental changes when their performance is affected [21]. The influence of environmental factors on plant growth can be either direct, via the impact of physical conditions on primary growth processes, or indirect due to developmental adaptation [25]. Plant growth is affected by numerous environmental factors, including water shortage and excess, temperature, nutrient availability, and light [26, 27]. Many plant traits are sensitive to climate [28]. Studies focusing on interspecific patterns between plant traits and climatic factors have identified a correlation between leaf area and mean annual precipitation (MAP) [29]. Variation in the leaf size and shape has been shown to be correlated with climatic factors [30]. In addition, other environmental factors, such as light intensity and nutrient availability, can influence leaf size and shape [31].

The relationships between functional leaf traits and climatic conditions have been emphasized for at least a century [32]. Leaf VLA usually increases with a decrease in average annual precipitation [33,34]. Leaf VLA and rainfall are strongly negatively related in evergreen shrubs and trees [32]. Jujube varieties growing in arid and semi-arid regions tend to have leathery and high-VLA leaves. Leaves with high leaf dry mass per area (LMA) usually have thick leaf blades, and small and thick-walled cells, which can adapt to very dry conditions [23].

Several studies have attempted to explain how plasticity and genotype affect the relationships between jujube tree traits and environmental factors [35–37]. Su and Liu [38] studied the photosynthetic characteristics of *linze* jujube under high temperature and irradiation. Cui et al. [39] investigated the response of vegetative growth, fruit development and water use efficiency of pear-jujubes to regulated water deficit at various growth stages and different levels of water deficit at a single growth stage. Gao et al. [40] studied the antioxidant capacity of different jujube cultivars grown in the Loess Plateau of China. Ma et al. [41] evaluated the effects of water deficit at different growth stages on pear-jujube trees. Cui et al. [42] pointed out that regulated water deficit, controlled by irrigation, could improve fruit quality and water use efficiency of pear-jujube trees. Cui et al. [42] also used the method of stable carbon isotope discrimination to study the water use efficiency of pear-jujube trees under regulated water deficit irrigation.

A clear understanding of vein traits of jujube leaves can help to understand and predict whole plant performance under different climatic conditions, with applications in breeding of improved jujube varieties [18]. An analysis of jujube leaf morphology under different climatic conditions can also improve our understanding of adaptive strategies of jujube in response to drought stress. However, few studies have been conducted on jujube leaf traits and their role in responding to climatic stresses. The sensitivity of leaf morphology of Chinese jujube to climate is generally poorly understood [5,41].

In this study, a survey and analysis has been conducted to investigate the relationship between jujube leaf morphology and climatic factors, across 33 sites in northern China. The objectives were (1) to determine how leaf venation traits of Chinese jujubes vary under different climatic conditions, especially under drought stress, (2) to quantify the relationships between functionally linked leaf traits and climatic factors, including mean annual temperature (MAT) and mean annual precipitation (MAP), and (3) to identify the similarities among jujube varieties grown at 33 sites in China.

## Materials and Methods

### 2.1 Study sites and leaf sampling

Leaf samples were collected mainly in private orchards, with the permission of land owners. We confirmed that the field studies did not involve any endangered or protected species.

**Fig 1:**
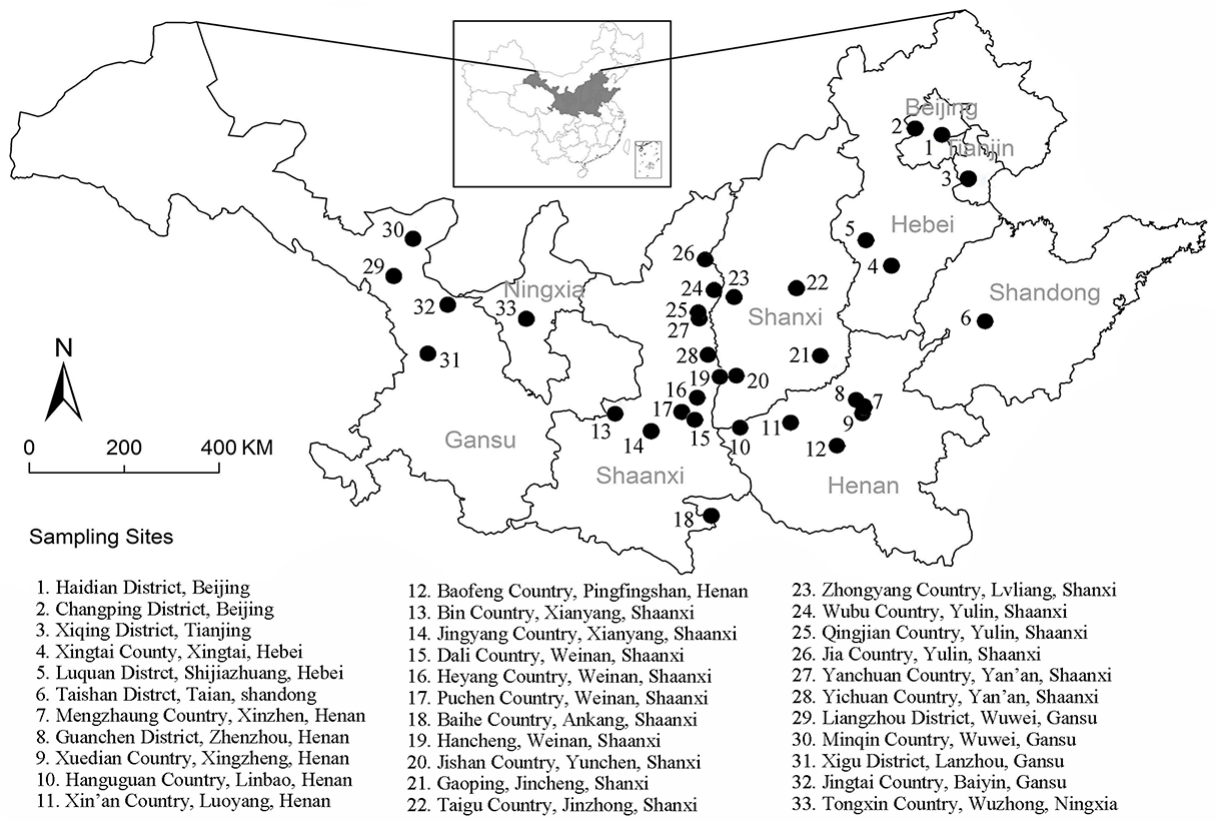
Distribution of the 33 sampling sites (counties or districts) of jujube leaves in northern China (34°–39° N and 102°–117° E; small map at top center). The dots represent the origin growth areas of the various jujube varieties where jujube leaves were collected.

Jujube leaf samples were collected in 33 sites in northern China in 2012 (Fig 1). The sampling sites above covered all of the three jujube production area in northern China, alluvial soils in the middle and lower reaches of the Yellow River and Haihe River (ASYH area), hills in the Loess Plateau (HLP area), and arid valleys and hills in Northwest China (AVHN area) [1]. The climates of these cultivation areas are very different, which provided a good opportunity to study the relationship between leaf morphology and environmental factors. The sampling sites covered all three jujube production areas.

The climates at the 33 study sites are generally temperate continental monsoon or temperate continental, with comparably low precipitation, and large diurnal and annual temperature differences. Winter periods are usually cold and dry, while summers periods are warm and wet. Moving closer to the center of continent, the climate becomes drier, with more frequent drought. For each sampling site, long-term climate data collected over 62 years (1951–2012), including MAT(°C) and MAP (mm), were obtained from the China Meteorological Data Sharing Service System (CMDSSS; http://cdc.cma.gov.cn/; Table 1). According to Table 1, Gansu and Ningxia Provinces tend to suffer from drought stresses since the average annual precipitations from the sampling sites in these provinces were all lower than 300 mm, while other provinces had much higher precipitations, usually larger than 500 mm. These two provinces are also prone to low temperature stresses since average annual temperatures were all below 10°C for their sampling sites.

**Table 1:** Geographic and climatic characteristics (1951–2012) of the 33 sampling sites for jujube leaves in northern China.

No.	Site (City, Province/ Municipality)	Latitude	Longitude	Average annual precipitation (mm)	Average annual air temperature (°C)	Minimum air temperature (°C)	Maximum air temperature (°C)
1	Haidian District, Beijing	N39°53'42"	E116°25'25"	581.8	12.4	-27.4	41.9
2	Changping District, Beijing	N40°11'55"	E116°04'15"	434.8	9.5	-26.2	39.2
3	Xiqing District, Tianjing	N38°59'30"	E117°30'14"	544.4	12.6	-22.9	40.5
4	Xingtai Country, Xingtai, Hebei	N37°06'22"	E114°12'45"	513.0	13.8	-22.4	42.4
5	Luquan District, Shijiazhuang, Hebei	N38°04'25"	E114°16'55"	524.3	13.5	-19.8	42.9
6	Taishan District, Taian, Shandong	N36°12'39"	E117°06'54"	688.9	12.8	-22.4	40.7
7	Mengzhuang country, Xinzheng, Henan	N34°35'11"	E113°49'07"	617.4	16.8	-7	42.9
8	Xuedian country, Xinzheng, Henan	N34°29'44"	E113°48'21"	708.3	15.4	-19.6	41.9
9	Guanchen District, Zhenzhou, Henan	N34°41'16"	E113°42'27"	619.0	14.5	-17.9	43.0
10	Lingbao, Sanmenxia, Henan	N34°37'04"	E110°55'19"	543.7	14.0	-16.5	43.2
11	Xin'an Country, Luoyang, Henan	N34°43'13"	E112°10'12"	582.7	14.8	-18.2	44.2
12	Baofeng Country,Pingdingshan, Henan	N33°52'26"	E113°03'07"	726.5	14.6	-19.1	43.4
13	Binxian country, Xianyang, Shaanxi	N35°01'22"	E107°59'29"	555.4	9.8	-26.2	37.6
14	Jingyang country, Xianyang, Shaanxi	N34°32'28"	E108°42'59"	550.0	13.0	-15.2	39.1
15	Dali country, Weinan, Shaanxi	N34°42'18"	E109°45'39"	630.8	13.5	-14.1	40.5
16	Heyang country, Weinan, Shaanxi	N35°19'18"	E110°21'03"	535.1	13.9	-13.5	39.5
17	Puchen country, Weinan, Shaanxi	N34°56'12"	E109°49'34"	515.1	14.2	-13.1	39.8
18	Baihe country, Ankang, Shaanxi	N32°45'05"	E109°46'08"	762.4	18.6	-13.7	20.3
19	Hancheng, Weinan, Shaanxi	N35°23'35"	E110°23'58"	577.0	14.0	-14.8	39.4
20	Jishan Country, Yuncheng, Shanxi	N35°38'50"	E110°50'39"	528.2	14.5	-18.9	42.7
21	Gaoping, Jincheng, Shanxi	N35°41'21"	E113°04'59"	572.4	10.1	-29.3	37.6
22	Jinzhong, Shanxi	N37°20'34"	E112°29'54"	452.9	10.9	-18.5	39.9
23	Zhongyang country, Lvliang, Shanxi	N37°21'10"	E111°05'52"	450.6	11.4	-24.5	40.6
24	Wubu country,Yulin, Shaanxi	N37°28´28"	E110°39´40"	433.9	10.8	-25.4	40.5
25	Qingjian country, Yulin, Shaanxi	N36°12'10"	E110°24'11"	510.2	10.0	-25.4	39.7
26	Jia country, Yulin, Shaanxi	N38°07´26"	E110°30´41"	387.9	8.5	-32.7	39.0
27	Yanchuan country, Yan'an, Shaanxi	N36°43´46"	E110°26´26"	496.8	10.0	-22.3	38.0
28	Yichuan country, Yan'an, Shaanxi	N36°14´20"	E110°07´45"	580.7	11.1	-23.0	37.5
29	Liangzhou District, Wuwei, Gansu	N38°41'24"	E103°04'48"	156.5	8.1	-32	40.8
30	Minqin country, Wuwei, Gansu	N38°30'52"	E102°58'46"	111.1	8.5	-29.5	35.9
31	Xigu District, Lanzhou,Gansu	N36°10'40"	E103°28'12"	308.2	9.6	-21.7	39.8
32	Jingtai country, Baiyin, Gansu	N37°07'38"	E104°19'54"	175.8	8.7	-27.3	39.4
33	Tongxin country, Wuzhong, Ningxia	N36°53'04"	E106°03'30"	257.8	9.0	-28.3	39.0

### 2.2 Leaf sample collection

Totally, leaf samples from 116 jujube varieties were collected from 33 the sites in northern China (S1 Table). The samples were collected during August 2012. The jujube varieties, representing the core collection in three cultivation areas, were analyzed for their leaf traits. The diameters at breast height (DBH) were 20–40 cm; the tree ages were over 20 years old. For each jujube variety, three representative trees were randomly selected. Five shedding shoots were sampled from both the exterior (‘sun leaves’) and interior (‘shade leaves’) canopies of five randomly selected trees from each jujube variety. Then, five leaves were randomly sampled from each shoot resulting in 25 leave samples per Jujube variety. The leaf samples were stored in a freezer at 4°C before processing.

### 2.3 Measurement of leaf morphologic parameters

Approximately three to five mid-leaves were selected from each shedding shoot to investigate vein visibility through chemical clearing [23]. All leaves were cleared using a protocol published previously [13,17,43]. Each leaf (excluding the petiole) was cut from the stem and gently patted dry before measuring leaf area [44]. Leaves were fixed in 70% formalin–acetic acid–alcohol (48% ethanol, 10% formalin, 5% glacial acetic acid, 37% water) and cleared in 2.5%–5% sodium hydroxide in water or ethanol, bleached with sodium hypochlorite, and stained with safranin and fast green [23].

Leaf area, perimeter, and vein density were measured using the ImageJ image analysis software (public software; http://rsb.info.nih.gov/ij/) [45]. Vein density was calculated as the sum of the lengths of all vein segments (mm) per unit area (mm^2^). An adaxial section of approximately one square centimeter at the right side of the midrib was excised from a sampled leaf to determine vein density. Leaf perimeter increased in proportion to the square root of leaf area for a given leaf type [46]. Leaf shape was recorded as perimeter^2^/area [47]. Since perimeter^2^/area was independent of leaf size, its mean value was calculated to represent all leaves sampled for each leaf type [48].

### 2.4 Data analysis

One-way analysis of variance (ANOVA) was performed on leaf trait data. A total of 21 representative jujube varieties from seven provinces were analyzed for their differences in leaf area, perimeter, and vein density through ANOVA. Then the differences of these leaf morphological traits were also analyzed for jujube varieties from three different jujube production areas, or ASYH, HLP, and AVHN areas mentioned previously. According to the Kolmogorov-Smirnov normality test (K–S test), VLA, leaf area, and leaf perimeter were normally distributed (p = 0.055, 0.200, and 0.200, respectively). Thus, post-hoc tests of VLA, leaf area, leaf perimeter could be conducted. Linear regression analysis was used to investigate potential relationships between leaf traits (e.g., vein density, leaf shape parameter of perimeter^2^/area) and environmental factors (e.g., mean annual precipitation and temperature). Finally, PCA and hierarchical clustering were performed on the morphological traits (VLA, leaf area, leaf perimeter, loopiness, distance between veins, number of nodes, and areole area) of the jujube leaves. The PCA results were used to describe plant trait and function types among populations via a covariance matrix, with data standardization [49]. For the statistical analyses described above, the SPSS (Statistical Product and Service Solutions) and SigmaPlot 11.0 (Systat Software, Richmond, CA, USA) software packages were used.

## Results

### 3.1 Influences of climatic factors on leaf morphology

**Fig 2:**
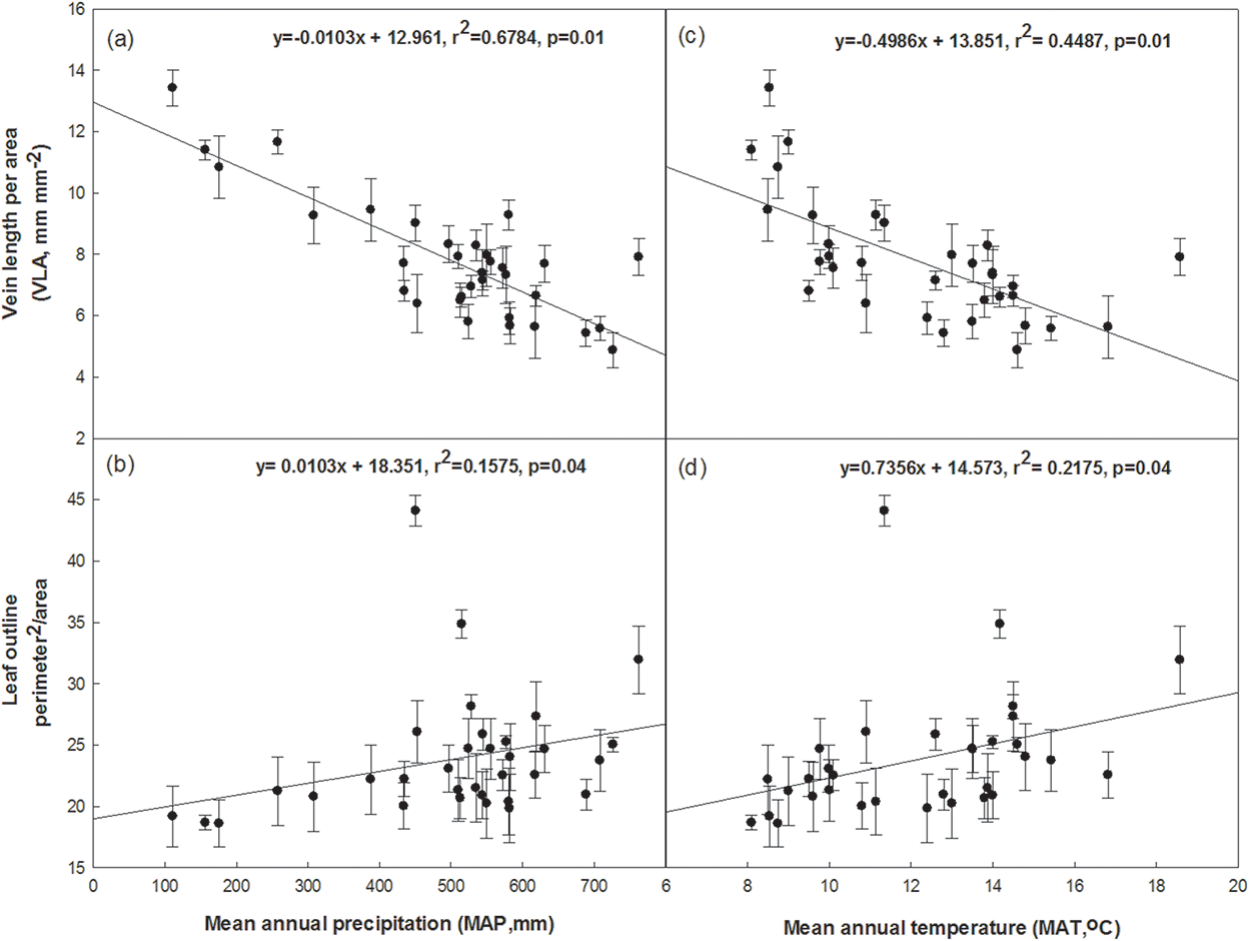
Relationships between jujube leaf traits of vein length per unit area (VLA), and leaf shape (perimeter^2^/area) and the climatic factors of mean annual precipitation (MAP) and mean annual temperature (MAT) in 33 sampling sites across northern China. Each dot represents 1of the 33 sampling sites in China. Error bars represent standard deviations.

Across the 33 sampling sites (Fig 1; Table 1), climatic conditions varied greatly, especially for MAT and MAP. The jujube leaves differed in size and shape due to environmental influences. VLA of jujube leaves had a negative linear relationship with MAP (r^2^ = 0.678, p = 0.01) and MAT (r^2^ = 0.449, p = 0.01), respectively (Fig 2A and 2C). Jujube leaf shape (perimeter^2^/area) significantly increased with MAP (r^2^ = 0.158, p = 0.04) and MAT (r^2^ = 0.218, p = 0.04; Fig 2B and 2D).

**Fig 3:**
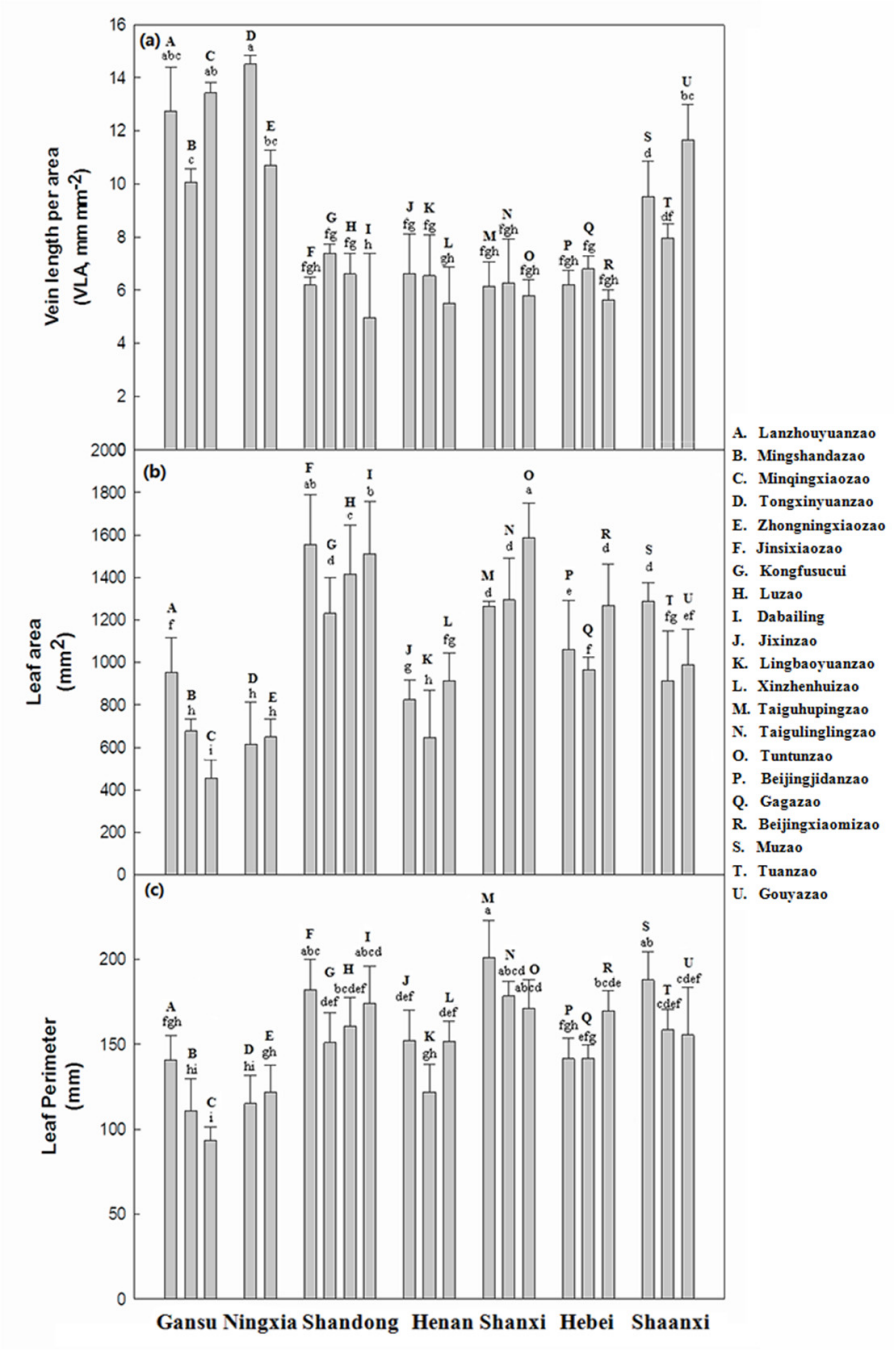
Comparisons of vein length per unit area (VLA) (a), area (b) and perimeter (c) of jujube leaves of 21 representative jujube varieties sampled in 33 sites in northern China. The jujube varieties are represented by different capital letters for the sake of clarity. The error bars are standard deviations of the variables concerned. The least significant difference (LSD) multiple comparison tests were conducted for VLA (df = 62, F = 21.10, p<0.05), leaf area (df = 62, F = 112.472, p<0.05), and leaf perimeter (df = 62, F = 8.016, p<0.05), respectively. Different lowercase letters above error bars for each component indicate statistically significantly different mean values (p<0.05).

MAP in the Gansu and Ningxia provinces was substantially lower than in other provinces, while MAT in the Gansu, Ningxia and Shaanxi provinces was slightly lower than those in other provinces (Table 1). Jujube from the Gansu, Ningxia and Shaanxi provinces had higher VLA values and lower leaf area and perimeter, particularly in Gansu. At the same time, the jujube varieties in the Shandong, Henan, Shanxi and Hebei provinces had relatively lower VLA, and higher leaf area and perimeter (Figs 2 and 3). Thus, it can be generally concluded that jujube grown in regions with lower MAP usually had higher VLA, and lower leaf area and perimeter.

### 3.2 Comparisons of leaf morphologic traits

Most of the morphologic traits, including VLA (Fig 3A), leaf area (Fig 3B), and leaf perimeter (Fig 3C) varied among the 21 jujube varieties representing the 33 testing sites in northern China. VLA presented significantly higher values in *Z*. *jujuba Mill*. cv. Tongxinyuanzao from Ningxia Province and the lowest was for *Z*. *jujuba Mill*. cv. Dabailing from the Shandong Province (Fig 3A). Leaf area presented significantly higher values in the variety *Z*. *jujuba Mill*. cv. Tuntunzao from Shanxi Province and significantly lower values in *Z*. *jujuba Mill*. cv. Minqinxiaozao from Gansu Province (Fig 3B). Leaf perimeter presented significantly higher values in the variety *Z*. *jujuba Mill*. cv. Taiguhupingzao from Shanxi Province and significantly lower values in *Z*. *jujuba Mill*. cv. Minqinxiaozao from Gansu Province (Fig 3C). Average annual rainfall in Gansu and Ningxia was markedly lower than rainfall in the other provinces (p<0.05), while average annual temperature in Gansu, Ningxia, and Shaanxi provinces was slightly lower compared to the remaining provinces. Thus, it could be generally concluded that jujube varieties from dry regions usually had lower leaf area and perimeter, and higher, than varieties from humid regions.

### 3.3 Similarities of leaf venation characteristics among different jujube varieties

**Fig 4:**
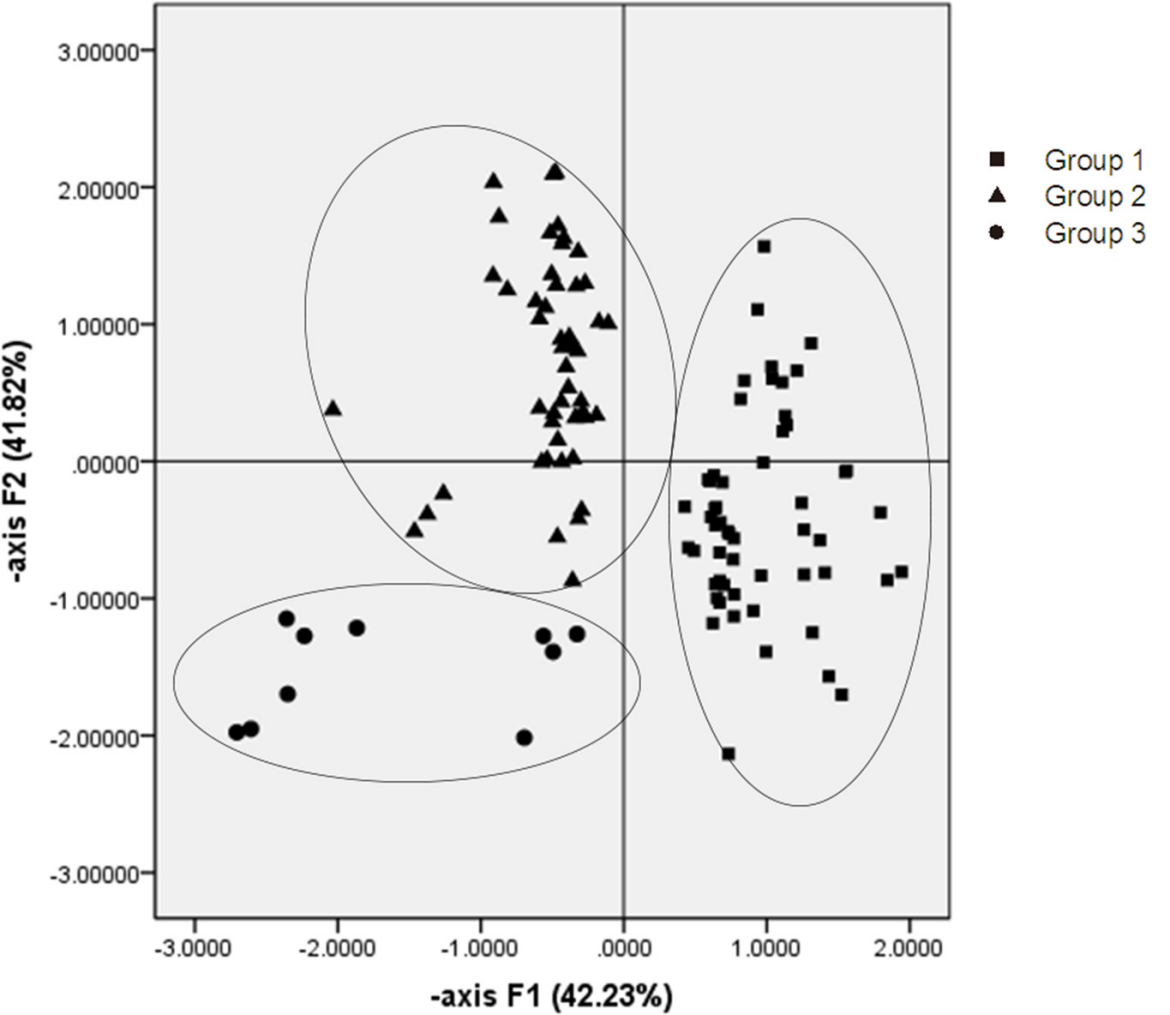
Principal components analysis of leaf morphological traits of 116 jujube varieties from 33 sites in northern China. All jujube varieties are divided into three groups: GROUP 1 (filled squares), GROUP 2 (filled triangles up), and GROUP 3 (filled circles). The seven provinces where the 33 sampling sites are located are represented by numbers for the sake of brevity. Members of GROUP 1 were mainly from Hebei, Shandong, Henan, southern Shanxi and central Shaanxi Provinces; Members of GROUP 2 were mainly from northwest Shanxi and northeast Shaanxi. Members of GROUP 2 were mainly from Gansu and Ningxia Provinces.

PCA results for plant leaf traits reflected morphological similarities among the 116 jujube varieties investigated (Fig 4). The first two axes accounted for 84.05% of the variability of leaf morphological traits. Hierarchical clustering divided the jujube varieties into three groups. Group 1 included mainly jujube varieties from Hebei, Shandong, Henan, southern Shanxi and central Shaanxi provinces. Members of Group 2 were mainly from northwest Shanxi and northeast Shaanxi, or along the Yellow River Canyon. Group 3 included mainly the jujube varieties from Gansu and Ningxia provinces, which possessed varying degrees of drought tolerance.

**Fig 5:**
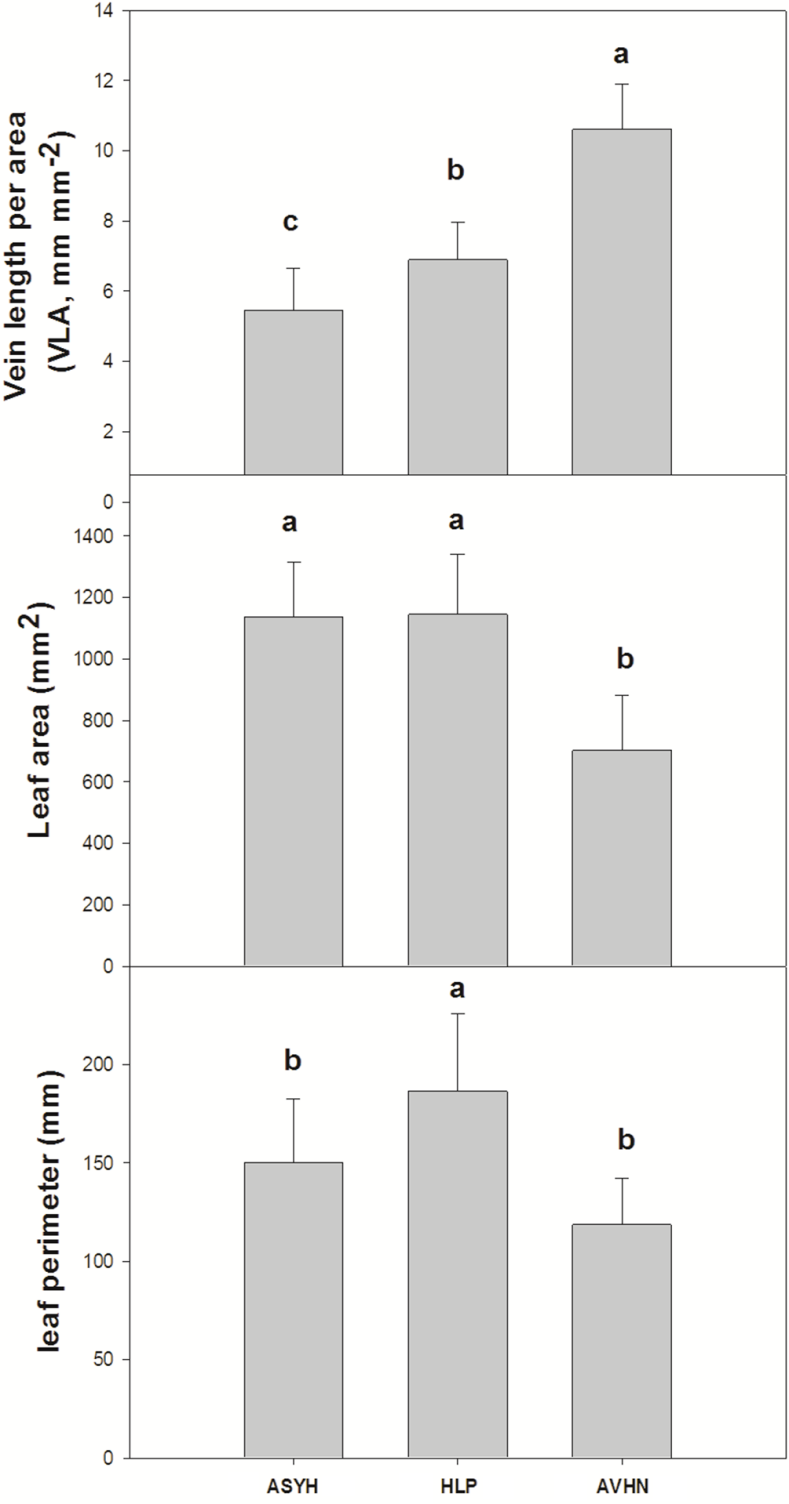
Comparisons of vein length per unit area (VLA), leaf area and leaf perimeter of jujube leaves sampled in three jujube cultivation areas (ASYH, HLP, and AVHN area) in China. The error bars are standard deviations of jujube production area concerned. (a) VLA (df = 35, F = 60.702, P<0.05); (b) leaf area (df = 35, F = 22.650, P<0.05); (c) leaf perimeter (df = 35, F = 8.726, P<0.05), as determined by LSD multiple comparison tests.

VLA, leaf area and leaf perimeter differed significantly among the growth habits. VLA presented significantly higher values in the jujube variety from the AVHN area and significantly lower values from the ASYH area (df = 35, F = 60.702, P<0.05; Fig 5A). Leaf area presented significantly lower values in the jujube variety from the AVHN area and significantly higher values from the HLP area (df = 35, F = 22.650, P<0.05; Fig 5B). Leaf perimeter presented significantly higher values in the jujube variety from the HLP area (df = 35, F = 8.726, P<0.05; Fig 5C).

## Discussion

### 4.1 Influences of climate on jujube leaf area

Interspecific variation in leaf area was related to climate. All of heat, cold, drought, nutrient and high-radiation stresses contribute to development of jujube trees with relatively small leaves [44]. Water and temperature are among the factors with the greatest impact on jujube leaf size [2]. The variety *Z*. *jujuba* Mill. cv. Minqinxiaozao from Ningxia Province had smaller leaf area and perimeter, as it often grew under drought and nutrient stress conditions. The variety *Z*. *jujuba* Mill. cv. Tuntunzao from Shanxi Province had larger leaf area and perimeter, as water and nutrients were sufficient in this province. This finding is similar to Li and Bao [50], who reported that jujube plants growing in drier sites have higher VLA. These leaf morphological traits reflect a general trend in plant adaptation when water is limited [51].

Jujube perimeter^2^/area, which is an index of intrinsic or size-independent shape [52] was positively related to MAT (r^2^ = 0.112) and MAP (r^2^ = 0.040), respectively (Fig 2B and 2D). Royer et al. [30] reported a significant linear relationship between perimeter/area and MAT within four jujube varieties in the eastern USA. The sizes and shapes of leaves were strongly linearly related to temperature and rainfall. There are biological bases for these relationships [52–55]. Warmer leaf temperatures promote both photosynthesis and transpiration [56]. VLA, leaf area and leaf perimeter of jujube from the Gansu and Ningxia provinces compared to other provinces (P<0.05; Fig 3). Jujube in the Gansu and Ningxia Provinces (arid valleys and hills), where the climate is dry and cool, tended to have smaller leaves to reduce evaporation, while larger leaves were more common in more humid areas such as the Shandong and Henan provinces. VLA, leaf area and leaf perimeter from the northwest Shanxi and northeast Shaanxi provinces were not remarkably different from the Shandong, Hebei, and Henan provinces (p>0.05; Fig 3). This might be because southern Shanxi and central Shaanxi provinces have alluvial soils in the middle and lower reaches of the Yellow River and Haihe River, while northeast and northwest Shanxi have hills in the Loess Plateau [1].

Since external factors, such as temperature and light regimes, fluctuate strongly, the impact on leaf growth could adversely affect leaf shape [24]. The lamina perimeter^2^/area is an index of leaf shape. All mesophyll regions of leaves with higher perimeter^2^/area will be closer to the veins [57]. Furthermore, leaves with higher perimeter/area tend to have a thinner boundary layer over the bulk of the lamina, which can enhance convective cooling and gas exchange at low wind speeds [48]. This can partially explain the finding that perimeter^2^/area increased with mean annual temperature and precipitation.

### 4.2 Relationship between VLA of Chinese jujube and drought tolerance

VLA was negatively and linearly related to MAP and MAT at the centers of origin of Chinese jujube (Fig 2). The negative relationship between vein density and MAP was also reported in other studies [13,14,58]. Leaf properties, such as vein density, are strongly related to the hydraulic conductivity of leaves [12]. The venation network is a key limiter of the hydraulic proficiency of angiosperm plants [12]. Vein traits are thought to reflect the gas and water exchange characteristics between leaves and the atmosphere, which are greatly influenced by climatic factors on the leaf, tree, stand, and even regional scales [58–60].

VLA can be used as an indicator of adaptation of jujube varieties to the local climate and habitat [58]. Since water supply must match transpiration demand of plants, VLA and stomatal pore area per leaf area tend to be positively related [9, 47]. A higher VLA can increase leaf xylem hydraulics, as it corresponds to a larger number of xylem flow pathways in parallel and a greater surface area of bundle sheaths and, thus, higher total permeability for water flow out of the veins [14, 16]. Higher VLA can improve leaf life span by providing redundant pathways around damaged sites or embolism during drought [32]. Thus, plants in dry regions tend to have higher VLA, which might lead to a longer leaf life span due to both biomechanical and hydraulic effects [18]. The resistance mechanisms of jujube in response to water stress can be explained in part through the leaf and water relationship [2].

The differences in other morphological traits of jujube leaves might also be caused by other regional environmental factors, such as soil fertility, thermal seasonality, and/or phylogenetic differences. Huff et al. [61] and Royer et al. [62] found that morphology of leaves from cold climates was consistent with the ecophysiological principles. A well-known generalization is that fast-growing, resource-acquisitive species tend to have lower LMA, higher light-saturation rates of photosynthesis per mass (A_mass_), higher N concentration per mass (N_mass_) and respiration rate per mass (R_mass_), but shorter leaf lifespan (LL), in contrast to slow-growing, resource-conservative species [20]. Thus, the jujube varieties in the drier Gansu and Ningxia provinces had relatively higher LMA, lower N_mass_ and R_mass_, but longer LL, in contrast to the jujube varieties in other humid provinces such as Henan, Shanxi and Shandong.

### 4.3 Adaptation of Chinese jujubes to local climatic conditions

Most of the jujube varieties investigated can grow well in their respective areas of origin. For instance, *Z*. *jujuba* Mill. cv. Lanzhouyuanzao and *Z*. *jujuba* Mill. cv. Tongxinyuanzao grow well and produce high yields in the Gansu and Ningxia provinces, where mean annual precipitation are only 156.5 and 257.8 mm, respectively. In this study, the highest values of VLA were found in the jujube varieties in these areas, which implies that VLA is a result of interactions between plant and environmental factors and can be used to reflect changes in the jujube plant morphology in response to environmental factors [63]. Uhl [58] also pointed out that VLA is a morphological characteristic that can adapt to changing environments. Thus, based on the analysis of VLA values, it can be concluded that jujube varieties in Gansu and Ningxia provinces have gradually adapted to their local climatic conditions of low temperature, high irradiance, and limited rainfall. This conclusion is consistent with some recent findings that leaf traits of Chinese jujubes have adapted to their local environments in the course of long-term evolution, embodying the ecological strategy of Chinese jujubes for drought resistance under the pressure of natural selection [4,38,39].

### 4.4 Introduction of jujube varieties based on vein traits

PCA divided the 116 jujube varieties into three different groups, according to leaf traits (Fig 4). Jujube varieties from Hebei, Shandong, Henan, and central Shaanxi provinces were similar and included in Group 1. Similarly, varieties from northwest Shanxi and northeast Shaanxi were similar and assigned to Group 2. Varieties from Gansu and Ningxia provinces were similar, comprising Group 3. This clustering result was consistent with the division of cultivation areas of Chinese jujubes [1]. In general, there were three jujube areas in China based on local natural conditions, including the area of alluvial soils in the middle and lower reaches of the Yellow River and Haihe River (ASYH area), the area of hills in the Loess Plateau (HLP area), and the area in arid valleys and hills in Northwest China (AVHN area). Interestingly, Hebei, Shandong, Henan, and central Shaanxi provinces all belonged to the ASYH area. Northwest Shanxi and northeast Shaanxi belonged to the HLP area, and Gansu and Ningxia provinces were in the AVHN area. Thus, within the context of leaf vein characters, the division of jujube cultivation areas was appropriate.

In China, introduction of new jujube varieties into some areas should take into consideration several ecological conditions, including temperature, precipitation, latitude, solar radiation, and altitude. The smaller the ecological differences, the easier the introduction of a new variety [1]. In this study, for jujube varieties from Gansu and Ningxia Provinces (Group 3 in Fig 4), there were no significant differences among the leaf morphological traits, as was the case for varieties from the Henan, Hebei, and Shandong provinces. The climatic conditions in Ningxia and Gansu were similar. Actually, for a long period, many jujube varieties from Gansu Province were introduced to Ningxia Province. The results of PCA of leaf venation characteristics partially showed the flow of jujube varieties among the provinces of China. Thus, when introducing proper jujube varieties, leaf venation characteristics should be taken into consideration. The jujube varieties that have leaf VLA, area, and perimeter similar to existing local varieties, might adapt more easily to the new environment.

## Conclusions

Jujube is a drought-tolerant tree species stretching across a variety of climatic conditions in China. This study summarized the survey results of 116 varieties of Chinese jujube grown in different environments across 33 sites in China. The results show that some important characteristics of jujube leaf morphology are linearly related to climatic factors such as MAT and MAP. Under drought stress, Chinese jujube tends to have higher VLA, and lower leaf area and leaf perimeter than varieties from humid regions. VLA was one of the key anatomical traits closely related to jujube transpiration and photosynthesis. VLA values varied among the sampling sites and were sensitive to climate. There was a linear relationship between jujube leaf VLA and the climatic factors MAP and MAT. By contrast, the shapes of jujube leaves (represented by leaf outline permeter^2^/area) were largely insensitive to MAT and weakly, linearly related to MAP.

Jujube from the Gansu, Ningxia and Shaanxi provinces had relatively higher VLA values and lower leaf area and perimeter. This was particularly the case for varieties from Gansu Province. Jujube varieties from Shandong, Henan, Shanxi and Hebei provinces had relatively lower VLA and higher leaf area and perimeter. According to the analysis of leaf morphological traits, different jujube varieties have gradually adapted to the local climatic conditions in their areas of origin. The jujube varieties were similar in morphology when grown under similar environmental conditions.

The results of PCA of leaf venation characteristics of 116 jujube varieties confirmed that the division into three main jujube cultivar areas in northern China is reasonable. Leaf morphological traits might be used as reference indices for jujube introduction between different areas. Generally, the jujube varieties with leaf VLA, area, and perimeter similar to existing local varieties might be easier to introduce.

## Supporting Information

S1 Table Jujube varieties studied for leaf morphological traits in this study. A total of 116 different jujube varieties were sampled in 33 sites in northern China.
(DOC)
