# Supplementary material for: Influences of Environmental Factors on Leaf Morphology of Chinese Jujubes
Source: PLoS One. 2015 May 28;10(5):e0127825. doi: 10.1371/journal.pone.0127825 (PMC4447434; doi:10.1371/journal.pone.0127825)
Supplement: S1 Table — A total of 116 different jujube varieties were sampled in 33 sites in northern China. (DOC) [file pone.0127825.s001.doc]

S1 Table. Jujube varieties studied for leaf morphological traits in this study. A total of 116 different jujube varieties were sampled in 33 sites in northern China.

| No. | Analysis name | Accession | Sampling site  （County, City, Province） | Geographical coordinate |
| --- | --- | --- | --- | --- |
| 1 | SXXBZ1 | Z.jujuba Mill. cv. Banzao | Taigu Country,Jinzhong, Shanxi | E112°29'54", N37°20'34" |
| 2 | SXXBDX1 | Z.jujuba Mill. cv. Baodexiaozao | Taigu Country,Jinzhong, Shanxi | E112°29'54", N37°20'34" |
| 3 | SXXBDYZ1 | Z.jujuba Mill. cv. Baodeyouzao | Taigu Country,Jinzhong, Shanxi | E112°29'54", N37°20'34" |
| 4 | BJJD1 | Z.jujuba Mill. cv. Beijingjidanzao | Haidian District, Beijing | E116°13'35", N39°58'28" |
| 5 | BJMZ1 | Z.jujuba Mill. cv. Beijingmizao | Haidian District, Beijing | E116°13'35", N39°58'28" |
| 6 | SXXBB1 | Z.jujuba Mill. cv. Benzao | Taigu Country,Jinzhong, Shanxi | E112°29'54", N37°20'34" |
| 7 | SXXBXJZ1 | Z.jujuba Mill. cv. Bingxianjinzao | Bin Country,Xianyang, Shaanxi | E107°59'29", N35°01'22" |
| 8 | SDDBL1 | Z.jujuba Mill. cv. Dabailing1 | Taishan District, Taian, Shandong | E117°07'09", N36°12'18" |
| 9 | SDDBL2 | Z.jujuba Mill. cv. Dabailing2 | Luquan District, Shijiazhuang, Hebei | E114°16'55", N38°04'25" |
| 10 | SXXDCZ1 | Z.jujuba Mill. cv. Dacuizao | Taigu Country,Jinzhong, Shanxi | E112°29'54", N37°20'34" |
| 11 | SXDLJD1 | Z.jujuba Mill. cv. Dalijidanzao | Dali Country, Weinan, Shaanxi | E109°45'39", N34°42'18" |
| 12 | SXDLLL1 | Z.jujuba Mill. cv. Dalilinglingzao | Dali Country, Weinan, Shaanxi | E109°45'39", N34°42'18" |
| 13 | SXDLMY1 | Z.jujuba Mill. cv. Dalimayazao | Dali Country, Weinan, Shaanxi | E109°45'39", N34°42'18" |
| 14 | SXDLMZ1 | Z.jujuba Mill. cv. Dalimianzao | Dali Country, Weinan, Shaanxi | E109°45'39", N34°42'18" |
| 15 | SXXDLOZ1 | Z.jujuba Mill. cv. Dalongzao | Taigu Country,Jinzhong, Shanxi | E112°29'54", N37°20'34" |
| 16 | SDFCM1 | Z.jujuba Mill. cv. Fucuimi | Taishan District, Taian, Shandong | E117°07'09", N36°12'18" |
| 17 | BJGGZ1 | Z.jujuba Mill. cv. Gagazao | Changping District, Beijing | E116°04'15", N40°11'55" |
| 18 | SXXGTZ1 | Z.jujuba Mill. cv. Guantanzao | Taigu Country,Jinzhong, Shanxi | E112°29'54", N37°20'34" |
| 19 | SXGDBZ1 | Z.jujuba Mill. cv. Cuicuizao | Wubu Country,Yulin, Shaanxi | E110°39´40", N37°28´28" |
| 20 | SXXHMZ1 | Z.jujuba Mill. cv. Hamazao | Taigu Country,Jinzhong, Shanxi | E112°29'54", N37°20'34" |
| 21 | SXHYL1 | Z.jujuba Mill. cv. Heyanglinglingzao | Heyang Country, Weinan, Shaanxi | E110°21'03", N35°19'18" |
| 22 | SXXHZS1 | Z.jujuba Mill. cv. Zhaohongshiyuehong | Taigu Country,Jinzhong, Shanxi | E112°29'54", N37°20'34" |
| 23 | SXXHPZ1 | Z.jujuba Mill. cv. Hupingzao | Taigu Country,Jinzhong, Shanxi | E112°29'54", N37°20'34" |
| 24 | SXHPZ2 | Z.jujuba Mill. cv. Baizao | Qingjian Country, Yulin, Shaanxi | E110°24´25", N37°04´37" |
| 25 | SXXHLZ1 | Z.jujuba Mill. cv. Huluzao1 | Taigu Country,Jinzhong, Shanxi | E112°29'54", N37°20'34" |
| 26 | HNHLZ2 | Z.jujuba Mill. cv. Huluzao2 | Mengzhuang Country, Xinzhen, Henan | E113°49'07", N34°35'11" |
| 27 | SXXHLCH1 | Z.jujuba Mill. cv. Huluchanghong | Taigu Country,Jinzhong, Shanxi | E112°29'54", N37°20'34" |
| 28 | HNJXZ1 | Z.jujuba Mill. cv. Jixinzao | Mengzhuang Country, Xinzhen, Henan | E113°49'07", N34°35'11" |
| 29 | SXXJZB1 | Z.jujuba Mill. cv. Jixianbanzao | Jishan Country,Yuncheng, Shanxi | E110°56'49", N35°38'18" |
| 30 | SXXJYZ1 | Z.jujuba Mill. cv. Jixianyuanzao | Jishan Country,Yuncheng, Shanxi | E110°56'49", N35°38'19" |
| 31 | BJJZ1 | Z.jujuba Mill. cv. Jiazao | Changping District, Beijing | E116°04'15", N40°11'55" |
| 32 | HNJTH1 | Z.jujuba Mill. cv. Jiantouhuizao | Guanchen District, Zhenzhou, Henan | E113°42'27", N34°41'16" |
| 33 | SXXJZ1 | Z.jujuba Mill. cv. Jianzao | Taigu Country,Jinzhong, Shanxi | E112°29'54", N37°20'34" |
| 34 | SXXJST1 | Z.jujuba Mill. cv. Jiaochentiansuanzao | Taigu Country,Jinzhong, Shanxi | E112°29'54", N37°20'34" |
| 35 | SXXJCY1 | Z.jujuba Mill. cv. Jinchangyihao | Taigu Country,Jinzhong, Shanxi | E112°29'54", N37°20'34" |
| 36 | SDJSY1 | Z.jujuba Mill. cv. Jinsiyihao | Taishan District, Taian, Shandong | E117°07'09", N36°12'18" |
| 37 | SDJSE1 | Z.jujuba Mill. cv. Jinsierhao | Taishan District, Taian, Shandong | E117°07'09", N36°12'18" |
| 38 | HBJSE2 | Z.jujuba Mill. cv. Jinsixiaozao | Xingtai Country, Xingtai, Hebei | E114°53'32", N37°42'39" |
| 39 | HBJXM1 | Z.jujuba Mill. cv. Jixianmuzao | LuquanDistrict, Shijiazhuang, Hebei | E114°16'55", N38°04'25" |
| 40 | SXXJUZ1 | Z.jujuba Mill. cv. Junzao | Taigu Country,Jinzhong, Shanxi | E112°29'54", N37°20'34" |
| 41 | SXXGS1 | Z.jujuba Mill. cv. Kashixiaozao | Qingjian Country,Yulin, Shaanxi | E110°04´53", N37°07´44" |
| 42 | SXGYZ1 | Z.jujuba Mill. cv. Gouyazao | Jia Country,Yulin, Shaanxi | E110°30´41, N38°07´26 |
| 43 | SDKFSC1 | Z.jujuba Mill. cv. Kongfucuisu | Taishan District, Taian, Shandong | E117°07'09", N36°12'18" |
| 44 | SXXKD1 | Z.jujuba Mill. cv. Kunduanzao | Taigu Country,Jinzhong, Shanxi | E112°29'54", N37°20'34" |
| 45 | SXXLJ1 | Z.jujuba Mill. cv. Lajiaozao | Taigu Country,Jinzhong, Shanxi | E112°29'54", N37°20'34" |
| 46 | GSLYZ1 | Z.jujuba Mill. cv. Lanzhouyuanzao1 | Liangzhou District, Wuwei,Gansu | E102°41'54", N38°03'41" |
| 47 | GSLYZ2 | Z.jujuba Mill. cv. Lanzhouyuanzao2 | Xigu District, Lanzhou,Gansu | E103°28'12", N36°10'40" |
| 48 | SXXLZ1 | Z.jujuba Mill. cv. Lizao | Taishan District, Taian, Shandong | E117°07'09", N36°12'18" |
| 49 | SXXLCDM1 | Z.jujuba Mill. cv. Lichendamazao | Taigu Country,Jinzhong, Shanxi | E112°29'54", N37°20'34" |
| 50 | SXXLFTZ1 | Z.jujuba Mill. cv. Linfentuanzao | Taigu Country,Jinzhong, Shanxi | E112°29'54", N37°20'34" |
| 51 | HNLBDZ1 | Z.jujuba Mill. cv. Linbaodazao | Lingbao, Sanmenxia, Henan | E110°55'19", N34°37'03" |
| 52 | HNLBYZ1 | Z.jujuba Mill. cv. Linbaoyuanzao | Hanguguan Country, Lingbao, Henan | E110°55'19", N34°37'04" |
| 53 | SXXLG1 | Z.jujuba Mill. cv. Liuguanzao | Taigu Country,Jinzhong, Shanxi | E112°29'54", N37°20'34" |
| 54 | HNLYX2 | Z.jujuba Mill. cv. Liuyuexian2 | Mengzhuang Country, Xinzhen, Henan | E113°49'07", N34°35'11" |
| 55 | HNLYX3 | Z.jujuba Mill. cv. Liuyuexian3 | Taishan District, Taian, Shandong | E117°07'09", N36°12'18" |
| 56 | HBLOZ1 | Z.jujuba Mill. cv. Longzao | Xingtai Country, Xingtai, Hebei | E114°53'32", N37°42'39" |
| 57 | SDLZY1 | Z.jujuba Mill. cv. Luzaoyihao | Taishan District, Taian, Shandong | E117°07'09", N36°12'18" |
| 58 | SDLZE1 | Z.jujuba Mill. cv. Luzaoerhao | Taishan District, Taian, Shandong | E117°07'09", N36°12'18" |
| 59 | SXMZZ1 | Z.jujuba Mill. cv. Mazizao | Yanchuan Country, Yan'an, Shaanxi | E110°26´26", N36°43´46" |
| 60 | HNMYT1 | Z.jujuba Mill. cv. Mayitou | Mengzhuang Country, Xinzhen, Henan | E113°47'15", N34°33'17" |
| 61 | MGDOZ1 | Z.jujuba Mill. cv. Mangguodongzao | Taigu Country,Jinzhong, Shanxi | E112°29'54", N37°20'34" |
| 62 | GSMQX1 | Z.jujuba Mill. cv. Mingqinxiaozao | Minqin Country, Wuwei, Gansu | E102°58'46", N38°30'52" |
| 63 | GSMSD1 | Z.jujuba Mill. cv. Mingshandazao | Liangzhou District, Wuwei, Gansu | E102°41'54", N38°03'41" |
| 64 | SXMGD1 | Z.jujuba Mill. cv. Mugedazao1 | Yanchuan Country, Yan'an, Shaanxi | E110°26´26", N36°43´46" |
| 65 | SXMGD2 | Z.jujuba Mill. cv. Mugedazao2 | Yichuan Country, Yan'an, Shaanxi | E110°07´45", N36°14´20" |
| 66 | SXMZ1 | Z.jujuba Mill. cv. Muzao | Yichuan Country, Yan'an, Shaanxi | E110°07´45", N36°14´20" |
| 67 | NHPG1 | Z.jujuba Mill. cv. Neihuangpingguo | Guanchen District, Zhenzhou, Henan | E113°42'27", N34°41'16" |
| 68 | SXCPZ1 | Z.jujuba Mill. cv. Bazhizao1 | Dali Country, Weinan, Shaanxi | E109°45'39", N34°42'18" |
| 69 | GSXKZ1 | Z.jujuba Mill. cv. Xiaokouzao | JingtaiCountry, Baiyin, Gansu | E104°19'54", N37°07'38" |
| 70 | SXXPYB1 | Z.jujuba Mill. cv. Pingyaobuluosu | Taigu Country,Jinzhong, Shanxi | E112°29'54", N37°20'34" |
| 71 | HBPGZ1 | Z.jujuba Mill. cv. Pingguozao | Luquan District, Shijiazhuang, Hebei | E114°16'55", N38°04'25" |
| 72 | SXXPP1 | Z.jujuba Mill. cv. Popozao1 | Taigu Country,Jinzhong, Shanxi | E112°29'54", N37°20'34" |
| 73 | SXXPP3 | Z.jujuba Mill. cv. Popozao3 | Gaoping, Jincheng, Shanxi | E112°49'10", N35°52'35" |
| 74 | PCZSZ1 | Z.jujuba Mill. cv. Puchenzhishezao1 | Puchen Country, Weinan, Shaanxi | E109°49'34", N34°56'12" |
| 75 | PCZSZ2 | Z.jujuba Mill. cv. Puchenzhishezao2 | Puchen Country, Weinan, Shaanxi | E109°49'34", N34°56'13" |
| 76 | SDJSXZ1 | Z.jujuba Mill. cv. Jinsixiaozao | Taishan District, Taian, Shandong | E117°07'09", N36°12'18" |
| 77 | SDLYX1 | Z.jujuba Mill. cv. Liuyuexian | Taishan District, Taian, Shandong | E117°07'09", N36°12'19" |
| 78 | SXPPZ1 | Z.jujuba Mill. cv. Shanxipopozao | Taigu Country,Jinzhong, Shanxi | E112°29'54", N37°20'34" |
| 79 | SXXSGD1 | Z.jujuba Mill. cv. Suangedazao | Bin Country, Xianyang, Shaanxi | E108°00'12", N35°01'46" |
| 80 | SXXTHP1 | Z.jujuba Mill. cv. Taiguhupingzao | Taigu Country,Jinzhong, Shanxi | E112°29'54", N37°20'34" |
| 81 | SXXLLZ1 | Z.jujuba Mill. cv. Taigulinglingzao | Taigu Country,Jinzhong, Shanxi | E112°29'54", N37°20'34" |
| 82 | SDTZTZ1 | Z.jujuba Mill. cv. Tengzhoutangzao | Taishan District, Taian, Shandong | E117°07'09", N36°12'18" |
| 83 | NXTXYZ1 | Z.jujuba Mill. cv. Tongxinyuanzao | Tongxin Country, Wuzhong, Ningxia | E106°03'30", N36°53'04" |
| 84 | SXXTTZ1 | Z.jujuba Mill. cv. Tuntunzao1 | Taigu Country,Jinzhong, Shanxi | E112°29'54", N37°20'34" |
| 85 | SXLNTZ1 | Z.jujuba Mill. cv. Lvnaitouzao | Jia Country,Yulin, Shaanxi | E110°40´52", N37°43´12" |
| 86 | SXXYBZ1 | Z.jujuba Mill. cv. Xiyingbenzao | Baihe Country,Ankang, Shaanxi | E109°46'08", N32°45'05" |
| 87 | SXXXFMZ1 | Z.jujuba Mill. cv. Xiangfenmuzao | Taigu Country,Jinzhong, Shanxi | E112°29'54", N37°20'34" |
| 88 | SXXXFYZ1 | Z.jujuba Mill. cv. Xiangfenyuanzao | Taigu Country,Jinzhong, Shanxi | E112°29'54", N37°20'34" |
| 89 | SXXYZ1 | Z.jujuba Mill. cv. Xiaoyuanzao | Yichuan Country,Yan'an, Shaanxi | E110°07´45", N36°14´20" |
| 90 | HNXZH1 | Z.jujuba Mill. cv. Xinzhenhuizao1 | Xuedian Country, Xinzheng, Henan | E113°48'21", N34°29'44" |
| 91 | HNXZH2 | Z.jujuba Mill. cv. Xinzhenhuizao2 | Xin'an Country, Luoyang, Henan | E112°08'08", N34°44'13" |
| 92 | HNXZH3 | Z.jujuba Mill. cv. Xinzhenhuizao3 | Baofeng Country, Pingdingshan, Henan | E113°03'11", N33°53'19" |
| 93 | SXDLZ1 | Z.jujuba Mill. cv. Dalongzao | Qingjian Country,Yulin, Shaanxi | E110°28´29", N37°05´46" |
| 94 | SXGXGD1 | Z.jujuba Mill. cv. Gouxigedazao | Jia Country,Yulin, Shaanxi | E110°40´46", N37°43´20" |
| 95 | HNXZJ1 | Z.jujuba Mill. cv. Xinzhenjidanzao | Xin'an Country, Luoyang, Henan | E112°08'08", N34°44'12" |
| 96 | HNXZXY1 | Z.jujuba Mill. cv. Xinzhenxiaoyuanzao | Mengzhuang Country, Xinzheng, Henan | E113°49'07", N34°35'11" |
| 97 | HBXT1 | Z.jujuba Mill. cv. Dongzao | Luquan District, Shijiazhuang, Hebei | E114°16'55", N38°04'25" |
| 98 | SXGTZ1 | Z.jujuba Mill. cv. Goutouzao | Yichuan Country,Yan'an, Shaanxi | E110°22´06, N36°52´12 |
| 99 | TJGGZ1 | Z.jujuba Mill. cv. Tianjingagazao | Xiqing District, Tianjing | E117°03'10", N39°03'40" |
| 100 | SXXXCT1 | Z.jujuba Mill. cv. Xuechentiaozao | Taigu Country,Jinzhong, Shanxi | E112°29'54", N37°20'34" |
| 101 | SXXYN1 | Z.jujuba Mill. cv. Yangnaizao | Hancheng, Weinan, Shaanxi | E110°34'02", N35°37'22" |
| 102 | SXXYXM1 | Z.jujuba Mill. cv. Yixianmuzao | Taigu Country,Jinzhong, Shanxi | E112°29'54", N37°20'34" |
| 103 | SXXYHM1 | Z.jujuba Mill. cv. Yongjihamazao | Taigu Country,Jinzhong, Shanxi | E112°29'54", N37°20'34" |
| 104 | SXYFS1 | Z.jujuba Mill. cv. Youfushuizao | Jingyang Country, Xianyang, Shaanxi | E108°42'59", N34°32'28" |
| 105 | SXXYCT1 | Z.jujuba Mill. cv. Yucituanzao | Taigu Country,Jinzhong, Shanxi | E112°29'54", N37°20'34" |
| 106 | SXXYYT1 | Z.jujuba Mill. cv. Yuciyazao | Taigu Country,Jinzhong, Shanxi | E112°29'54", N37°20'34" |
| 107 | HNYCTZ1 | Z.jujuba Mill. cv. Yongchentuanzao | Guanchen District, Zhenzhou, Henan | E113°42'27", N34°41'16" |
| 108 | SXXYC1 | Z.jujuba Mill. cv. Yuancuizao | Taigu Country,Jinzhong, Shanxi | E112°29'54", N37°20'34" |
| 109 | SDYLY1 | Z.jujuba Mill. cv. Yuanlingyihao | Taishan District, Taian, Shandong | E117°07'09", N36°12'18" |
| 110 | SXXYPP1 | Z.jujuba Mill. cv. Yunchengpopozao | Taigu Country,Jinzhong, Shanxi | E112°29'54", N37°20'34" |
| 111 | SDZHD1 | Z.jujuba Mill. cv. Zhanhuadongzao | Taishan District, Taian, Shandong | E117°07'09", N36°12'18" |
| 112 | SDCHZ1 | Z.jujuba Mill. cv. Changhongzao | Taishan District, Taian, Shandong | E117°07'09", N36°12'19" |
| 113 | NXZNTX1 | Z.jujuba Mill. cv. Zhongningxiaozao | Tongxin Country, Wuzhong, Ningxia | E105°47'30", N37°06'40" |
| 114 | NXZNYX | Z.jujuba Mill. cv. Zhongningyuanzao | Tongxin Country, Wuzhong, Ningxia | E105°47'30", N37°06'40" |
| 115 | SXXZYT1 | Z.jujuba Mill. cv. Zhongyangtuanzao | Zhongyang Country, Lvliang, Shanxi | E111°05'52", N37°21'10" |
| 116 | SXZZ1 | Z.jujuba Mill. cv. Zhuizao | Yanchuan Country,Yan'an, Shaanxi | E110°22´09", N36°51´59" |
